# Supplementary material for: Individual and population-level risk factors for new HIV infections among adults in Eastern and Southern Africa
Source: Nat Commun. 2026 Jan 6;17:1195. doi: 10.1038/s41467-025-67966-0 (PMC12858868; doi:10.1038/s41467-025-67966-0)
Supplement: Supplementary file 1 — Supplementary Information [file 41467_2025_67966_MOESM1_ESM.pdf]

# Supplementary Information:
# Individual and population-level risk factors for new sexually-transmitted HIV infections among adults in sub-Saharan Africa

## Contents

Supplementary Notes: Review of the literature ... 3

Supplementary Methods: Harmonisation of data ... 11

Supplementary Methods: Additional information on methods ... 13

Supplementary Results ... 14

- Flowcharts showing participation in incidence cohort ... 14
- Distribution of each risk factor by age, sex and study ... 18
- Summary of results for each risk factor ... 18
- Crude HR for HIV incidence for all risk factors in each study, for men and women by age ... 22
- Adjusted risk factor models for HIV incidence using all studies' pooled data ... 27
- Sensitivity analysis ... 28
- Models including measures of partnerships dynamics in the community ... 28

Supplementary References ... 30

## List of tables

Supplementary Table 1: Summary of risk factors identified in population-based studies of HIV incidence in sub-Saharan African countries ... 5

Supplementary Figure 1:Framework for analysis ... 10

Supplementary Figure 2: Chart showing the availability of selected items of data for each study and survey round ... 12

Supplementary Figure 3: Flowcharts for women and men in each study showing the number of people included for analysis in the incidence cohort. Numbers in incidence cohort include everyone who was included in at least one imputation. People could contribute data to both age groups and may therefore have been counted twice in the bottom row. ... 15

Supplementary Data 1: Distribution of person years by socio-demographic characteristics and sexual behaviour among the men in each study for whom there is information, as a percentage of the total time spent in each age group. ... 22

Supplementary Data 2: Distribution of person years by socio-demographic characteristics and sexual behaviour among the women in each study for whom there is information, as a percentage of the total time spent in each age group. ... 22

Supplementary Data 3: Crude HR for HIV incidence in each study among women aged 15-24 ... 26

Supplementary Data 4: Crude HR for HIV incidence in each study among women aged 25-49 ... 26

Supplementary Data 5: Crude HR for HIV incidence in each study among men aged 15-24 .............. 26

Supplementary Data 6: Crude HR for HIV incidence in each study among men aged 25-49 ............. 26

Supplementary Data 7: Adjusted HR for HIV incidence among women aged 15-24 from four multivariate models ........................................................................................................... 28

Supplementary Data 8: Adjusted HR for HIV incidence among women aged 25-49 from four multivariate models ........................................................................................................... 28

Supplementary Data 9: Adjusted HR for HIV incidence among men aged 15-25 from four multivariate models ........................................................................................................... 28

Supplementary Data 10: Adjusted HR for HIV incidence among men aged 25-49 from four multivariate models ........................................................................................................... 28

Supplementary Table 2: Results from leave-one-out meta-analysis to assess the influence of individual studies. Lowest and highest HR obtained and the pooled HR estimated by meta-analysis of all study specific estimates. ........................................................................................ 29

Supplementary Data 11: Adjusted HR for selected risk factors for women in Karonga, Kisesa and Rakai including measures of partnership dynamics, by age for 2005-16 inclusive ............................ 30

Supplementary Data 12: Adjusted HR for selected risk factors for men in Karonga, Kisesa and Rakai including measures of partnership dynamics, by age for 2005-16 inclusive ...................................... 30

Supplementary Data 13: Adjusted HR for selected risk factors for 2013-16 only, based on data from Kisesa, Masaka, Rakai, uMkhanyakude, Kisumu and Ifakara.............................................................. 30

# Supplementary Notes: Review of the literature

The initial literature review was undertaken in 2016 and updated in 2023. We looked for papers which described estimates of effect size for factors associated with incident HIV infection in members of the general population living in sub-Saharan African countries with no exclusion based on study or publication date. We searched in Pubmed, Embase, Popline and African Index Medicus (AIM).

A total of 3403 titles and abstracts were screened and 40 full text papers were reviewed. We excluded papers where: the primary outcome was not HIV incidence, no empirical data were reported, no risk factors were described, the study was not population-based, or situated in sub-Saharan Africa.. Following full text review, we included 30 studies. One extra study, for Manicaland, was obtained from searching the references of the retrieved papers[1] resulting in 31 papers. The characteristics of each study are shown in S Table 1 together with a summary of the associations identified. Socio-demographic risks, such as age or place of residence have been omitted since these are context-specific and are likely to be important only as proxies for more proximate determinants of risk.

Many used data from ALPHA member studies with the most based on data from Rakai (N=14) [2-15], followed by five from Manicaland [1,16-19], three from uMkhanyakude[20-22], two using Masaka data[23,24] and one from Kisesa[25]. The remaining non-ALPHA studies were from South Africa[26,27] Uganda[28-30], and a microbicide trial pilot[31]. Nineteen of the 31 studies were from Uganda, with five each from South Africa and Zimbabwe and just one from Tanzania. There is a therefore a lack of geographic representation in the literature, with many countries omitted entirely and just one nationally-representative study.

The existing analyses from ALPHA member studies were for different time periods, covered slightly different age groups and found some contrasting associations. Similar discrepancies were observed across all the papers reviewed.

The Rakai papers covered the period 1994 to 2011 and consistently found associations between HIV incidence and marital status, alcohol use, inconsistent use of condoms, genital warts, genital ulcers and having multiple partnerships. Circumcision was also found to be protective in later studies, and intimate partner violence a risk. Kagaayi et al[4] found that community prevalence of HIV among the opposite sex and having a new partner were risks for incident HIV. The study with the most similar approach to Kagaayi et al is Nalugoda et al[9] who found that that the effect of marriage differed for men and women (current marriage was protective for women and former marriage was a risk for men) and that having more than one partner was a risk.

In neighbouring Masaka, genital discharge and syphilis were identified as risks in one study[24] and a lack of circumcision (proxied by religion) and large age gaps between partners were risks in another[23].

Elsewhere in Uganda, in the fishing communities around Lake Victoria, alcohol use was identified as a risk by Kiwanuka et al[28] and Seeley et al[30] with the latter also finding associations with religion, time living in the community, genital discharge or sores in past 3 months; smoking and marijuana use.

A Ugandan national survey in  found, for both sexes combined, that HIV incidence risk was increased by being formerly married compared to never married, having multiple partnerships, HSV-2 infection, STI symptoms in last year, and not being circumcised[29].

The South African studies also found associations with marriage[20-22,27], and single studies identified risk associated with inconsistent condom use[21], STIs[27] and multiple partnerships[22]. Two studies found associations with coital frequency[26,27] and other studies found associations with employment, wealth, age gap between partners, education and migrant status. In one study, community estimates of men's lifetime partners and HIV prevalence was associated with women's seroconversion risk[22].

In Zimbabwe, two analyses of Manicaland data showed associations with multiple partnerships for both sexes[1,16]. Lopman et al found that genital sores and having an unwell partner were also important for both men and women, with community HIV prevalence an important risk for men, and thinking the partner had other partners important for women[1]. In another analysis, community group membership was protective for women, but not for men[16].

Overall, the most commonly identified associations were with marriage in five populations[2,4,7,9-11,15,21,22,29], multiple partners in four populations[1,4,9-11,15,16,22,29], STIs in six populations[1,2,4,5,7,10,12,24,27,29-31] and alcohol use in two populations[2,4,7,10,11,15,28]. The associations seen with STIs could be causal but a number of authors ascribed the associations to coincidence, due to a set of common risk factors and mode of transmission. The marital status associations varied between studies, perhaps in part due to differences in the comparisons made, and also by sex where analyses were done separately for men and women.

Many studies analysed men and women together, controlling for sex, presumably to maximise the power available for the analysis. This precluded identifying the effects of risks which were different for men and women.

Some of the studies were quite small for multivariate analysis of HIV incidence in communities where incidence rates were around 1/100 person years. Though most did not present power calculations, it seems likely that several lacked the power to detect some of the observed risk factor associations and therefore it is difficult to interpret a lack of association as evidence of no association for these populations.

The existing literature does not therefore present a clear or consistent picture of the important risk factors for HIV incidence among men and women in sub-Saharan African countries.

Supplementary Table 1: Summary of risk factors identified in population-based studies of HIV incidence in sub-Saharan African countries.

| Country | First author (publication year) | Area | ALPHA study | Sex | Age range | Time period | Scope | Factors associated with HIV incidence |
|---|---|---|---|---|---|---|---|---|
| **UGANDA: RAKAI** | | | | | | | | |
| Uganda | Santelli (2015)[11] | Rakai | yes | M&F | 15-24 | 1999-2011 | Cohort of young people | Ever having been married was a risk for men and protective for women, more than one partner in the last year was a risk for women, alcohol in last month a risk for men. Being in school was protective for young women. |
| Uganda | Mathur (2015)[7] | Rakai | yes | M&F | 15-24 | 2005-2011 | Cohort of young people | Women: Formerly married a risk compared to never married, partner's alcohol use and inconsistent condom use also increased risk.<br>Men: current marriage and former marriage both riskier than never.<br>Both: non-marital partner riskier than marital, genital warts increase risk, |
| Uganda | Edelstein (2015)[2] | Rakai | yes | M&F | 15-24 | 1999-2008 | Sexually experienced young people | Men: former marriage risky compared to never married; alcohol use and genital ulcers also risky.<br>Women: current marriage protective and former marriage risky compared to never married; being a student lowered risk; multiple partners and STI symptoms increased risk |
| Uganda | Nalugoda (2014)[9] | Rakai | yes | M&F | 15-49 | 1999-2011 | Cohort | More than one partner was risky; current marriage was protective for women and former marriage was a risk for men; |
| Uganda | Kagaayi (2014)[4] | Rakai | yes | M&F | 15-49 | 2003-2011 | Sexually active cohort | Women: Never married and separated riskier than current marriage; New partner, alcohol use, genital ulcers, risk |

| | | | | | | | | |
|---|---|---|---|---|---|---|---|---|
| | | | | | | | | perception and community prevalence also associated with risk.<br>Men: Post-primary education protective; 3+ partners, genital ulcers, partner of unknown status, partners in high-risk occupations were risky. Circumcision was protective. |
| Uganda | Grabowski (2014)[3] | Rakai | yes | M&F | 15-49 | 2008-2009 | Cohort | Investigated spatial clustering, including associations between HIV status and that of household members and nearby residents plus the effect of geographic location of sexual partners. |
| Uganda | Wagman (2014)[13] | Rakai | yes | M&F | 15-49 | 2005-2009 | Intervention nested in cohort | The effect of observed risk factors on HIV incidence was not reported. |
| Uganda | Santelli (2013)[10] | Rakai | yes | M&F | 15-24 | 1999-2008 | Sexually active cohort | Compared to never married, formerly married was a risk for men and women and currently married women were at lower risk.<br>Men: Alcohol use and genital ulcers were risks<br>Women: STI symptoms (genital warts, vaginal itching and painful urination) and multiple partners were risks. Being a student was protective. |
| Uganda | Kouyoumdjian (2013)[6] | Rakai | yes | F | 15-49 | 2000-2009 | Cohort of women in a partnership(s) | Only looked at intimate partner violence and found it to be a risk |
| Uganda | Wawer (2009)[14] | Rakai | yes | F | 15-49 | 2003-2007 | Circumcision intervention trial | Stopped early, circumcision was not protective |
| Uganda | Tobian (2009)[12] | Rakai | yes | M | 15-49 | not stated | Circumcision intervention trial | Previously married, washing genitals, genital ulcers, urethral discharge and HSV-2 all associated with risk. Circumcision was protective. HSV-2 likely |

| | | | | | | | | |
|---|---|---|---|---|---|---|---|---|
| | | | | | | | | to be coincident with HIV infection and so not a risk. |
| Uganda | Kiwanuka (2009)[5] | Rakai | yes | M&F | 15-49 | 1997-2002 | Serodiscordant couples cohort | Genital ulcers and not using condoms increased risk. |
| Uganda | Matovu (2006)[8] | Rakai | yes | M&F | 15-49 | not stated | Cohort | No effect of VCT on incidence |
| Uganda | Zablotska (2006)[15] | Rakai | yes | M&F | 15-49 | 1994-2002 | Cohort | Men: more than one partner, being never married compared to married, no education compared to secondary, and alcohol use before sex increased risk. Women: alcohol use, multiple partners and inconsistent condom use were risks. Possession of transport was protective. |
| UGANDA: MASAKA | | | | | | | | |
| Uganda | Biraro (2013)[23] | Kyamulibwa GPC, Masaka District | yes | M&F | 18-59 | 1989-2007 | Serodiscordant married couples cohort | Non-Muslim religion (as a proxy for lack of circumcision) and a 15+ year age gap between partners were both risks. |
| Uganda | Ruzagira (2011)[24] | Masaka District | no | M&F | 18-60 | 2006-2009 | Serodiscordant couples cohort | Risks were genital discharge and syphilis. No seroconversions in couples that consistently used condoms. |
| | | | | | | | | |
| Uganda | Kiwanuka (2014)[28] | Lake Victoria | no | M&F | 18-49 | | Fishing community cohort | Both sexes combined: alcohol use a risk. No evidence for marriage, new partners, condoms, circumcision. |
| Uganda | Seeley (2012)[30] | Lake Victoria | no | M&F | 13-49 | 2009-2010 | Fishing community cohort | Risks for both sexes combined: Protestant or Muslim versus Catholic; arrived in community in the last 5 years; genital discharge or sores in past 3 months; alcohol use, smoking, marijuana use. Not associated: marital status, education level, ethnicity, and financial dependency, number of sexual partners in the previous 3 months, having sex under the influence of alcohol, |

| | | | | | | | | |
|---|---|---|---|---|---|---|---|---|
| | | | | | | | | knowledge of sexual partners with HIV infection, and being away from home frequently |
| UGANDA: NATIONAL | | | | | | | | |
| Uganda | Mermin (2008)[29] | national | no | M&F | 15-59 | 2005 | 2005 Uganda HIV/AIDS Sero-Behavioral Survey | Both sexes combined: formerly marriage compared to never married, multiple partnerships, HSV-2 infection, STI symptoms in last year, and not being circumcised increased risk. |
| SOUTH AFRICA: KWAZULU-NATAL | | | | | | | | |
| South Africa | Harling (2015)[21] | KwaZulu-Natal | yes | F | 30-50 | 2003-2012 | Cohort of women with partners | Marriage protective; inconsistent condom use risky; bigger age gap (older partner) protective. |
| South Africa | Tanser (2011)[22] | KwaZulu-Natal | yes | M&F | 15-55 | | | Community estimates of men's lifetime partners and HIV prevalence associated with women's seroconversion risk. Education and marriage were protective whilst more than one partner was a risk. |
| South Africa | Bärnighausen (2007)[20] | KwaZulu-Natal | yes | M&F | F: 15-47 M: 15-52 | 2003-2005 | Cohort | Both sexes combined: Middle wealth riskier than poorest, non-migrant riskier than migrant, sexually active & unmarried riskier than single. |
| SOUTH AFRICA | | | | | | | | |
| South Africa | Wand (2010)[27] | Durban | no | F | not stated | not stated | Three community cohorts | Unemployed, being single, high coital frequency, STIs and pregnancy all associated with incidence. |
| South Africa | Hargreaves (2009)[26] | rural South Africa | no | M&F | 14-35 | 2001-2004 | Community microfinance trial | Both sexes combined: lower risk with infrequent sex |
| TANZANIA | | | | | | | | |

| | | | | | | | | |
|---|---|---|---|---|---|---|---|---|
| Tanzania | Cawley (2014)[25] | Kisesa | yes | M&F | | 2003-2010 | Cohort | Only VCT as a risk factor |
| ZIMBABWE | | | | | | | | |
| Zimbabwe | Gregson (2013)[17] | Manicaland | yes | M&F | 15-54 | 1998-2008 | Cohort | Membership of community groups protective for women but not men. Size of effect changed over time. |
| Zimbabwe | Gregson (2011)[16] | Manicaland | yes | M&F | M 15-54<br>F 17-44 | 1998-2003 | Cohort | Multiple partners, casual sex without a condom increased risk.<br>Marriage protective for women. |
| Zimbabwe | Lopman (2008)[1] | Manicaland | yes | M&F | M 15-54<br>F 17-44 | 1998-2003 | Cohort | Men: multiple partners, genital sores, community HIV prevalence and having an unwell partner were risks. No evidence for condom use, circumcision, or type of partner.<br>Women: multiple partners, genital sores, having an unwell partner and thinking partner had other partners were risks. Community prevalence, condom use and types of partner were not important. |
| Zimbabwe | Gregson (2011)[16] | Manicaland | yes | M&F | M 15-54<br>F 17-44 | 1998-2003 | Cohort | Community group membership was protective for women but not men |
| Zimbabwe | Mundandi (2006)[19] | Manicaland | yes | M&F | M: 17-54<br>F: 15-44 | 1998-2003 | Cohort | Looked at HIV status as a risk factor for migration |
| MULTIPLE COUNTRIES | | | | | | | | |
| Zimbabwe and Malawi | Kumwenda (2006)[31] | Harare, Blantyre and Lilongwe | no | F | 18+ | 1999-2001 | Pilot for abandoned microbicide trial | Being unmarried versus married, having an STI or bacterial vaginosis all risks, plus an increase in risk with more education. |

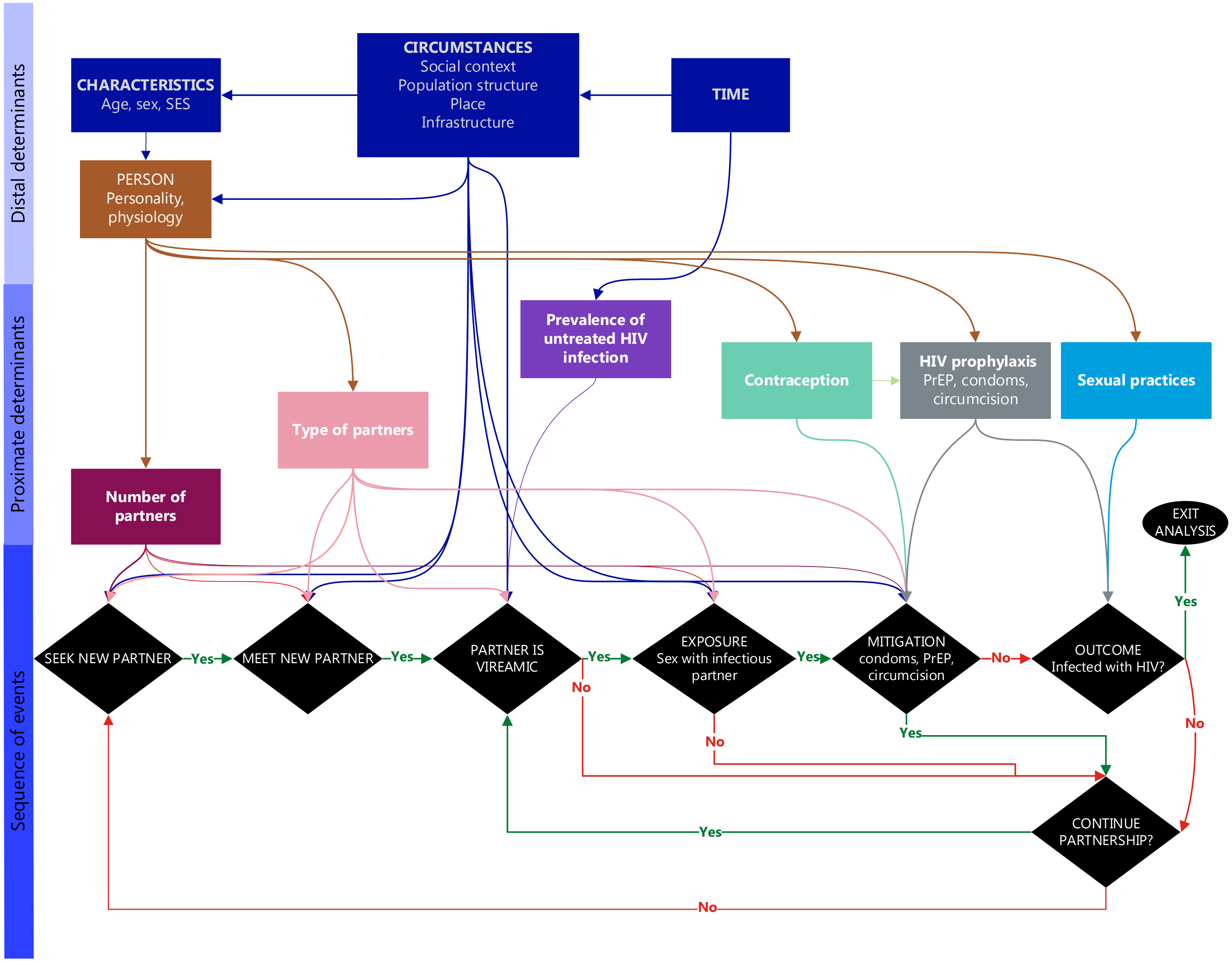


Supplementary Figure 1:Framework for analysis

# Supplementary Methods: Harmonisation of data

This is secondary analysis of data from diverse longitudinal studies. At the outset we collated and reviewed questionnaires for all the individual survey rounds carried out in each study and identified items relating to HIV risk factors. We then compiled a list of common measures which could be constructed for at least a portion of the follow up period in every study. Each study prepared their data to a common data specification (see https://alpha.lshtm.ac.uk/data-2/).

Data were available on residence, residential mobility, education, marital status, the start and end of partnerships, numbers and types of partnerships, age of partners, coital frequency, condom use and circumcision status. There was also information, either from only a few studies or for a short period only, on: sex in the last month, number of partners in last month, disclosure of HIV status to partners and by partners, circumstances of first sex, age at first sex, age at second sex, contraception, sexual violence and transactional sex. This latter information was not used for the pooled analysis.

Supplementary Figure 1 shows the percentage of study participants in each survey round for whom selected items of data were available. Where there were no data, this indicates that the information was not collected in that survey round. For each study and round where data were incomplete, we assessed the reasons for this and whether the missing data introduced any bias. Where this risk was minimal, we included the data from that round and categorised person time from those with missing data as unknown. Where there was likely to be a bias introduced by including the information, we discarded it for everyone. In some studies, there was partial information available before or after the main period of data collection. We discarded information before 2009 for Kisumu and after 2012 for Karonga and Manicaland because they did not actively collect data from the whole study population during those periods.

Table 1 (in the main paper) gives the definitions of the harmonised variables used in the analysis and how these correspond to the conceptual framework in Figure 1.

We did not impute missing data because we did not believe that would be unbiased. Instead, we have coded all instances where an item of data was not available, as “not available”, regardless of the reason. This may include rounds where questions were not asked, where respondents did not participate and questions that the respondent did not, or could not, answer. Most data were missing for the first two reasons. We believed the data to be missing not at random because the reasons for the data to be missing are linked to the HIV incidence. Information mising because questions were not asked is linked to time and place, which is also correlated with HIV incidence. Missing data that results from a respondent missing a round may also be associated with incidence. Reasons for non-participation are short-term migration, absence for work, or due to ill health or caring responsibilities and all of these may be associated with HIV incidence.

Some information was not available for all studies but we nevertheless decided to include it because they were thought to be important risk factors: coital frequency, age at circumcision and partnership dynamics.

Coital frequency was collected in four studies: Ifakara, Kisesa, Kisumu and Manicaland. Ifakara and Kisesa used the same questionnaire and Kisumu also used the same approach asking respondents for their coital frequency with each partner listed in the partner history in either the previous week, month or year depending on the timing of last sex. In Manicaland, respondents were asked for their coital frequency in the past two weeks with each partner. These responses were transformed into the number of acts per week, for ongoing partnerships only, and summed to give a total weekly frequency.

In most studies, circumcision was not asked about in the earlier surveys, particularly in places such as Karonga where men were not traditionally circumcised. When these data were collected it was more common to ask about current status than about age at circumcision. Where age at circumcision was asked it was possible to backdate circumcision status to that age, whereas current status data was limited to the time since the first report. When status changed between survey rounds, the status was changed at the date of the interview when circumcision was first reported.

Estimation of partnership acquisition and loss rates requires the start and end dates of each partnership that occurred in the last year and whether it was ongoing at the time of the survey. These were collected for sufficient respondents and the data were of adequate quality in Karonga, Kisesa and Rakai. Methods for calculating partner acquisition rates have been described elsewhere[25].

In one survey in Kisesa, marital status was ascertained only as ever or never married so it is not possible to identify people who are currently married for a short period of follow up.

Supplementary Figure 2: Chart showing the availability of selected items of data for each study and survey round

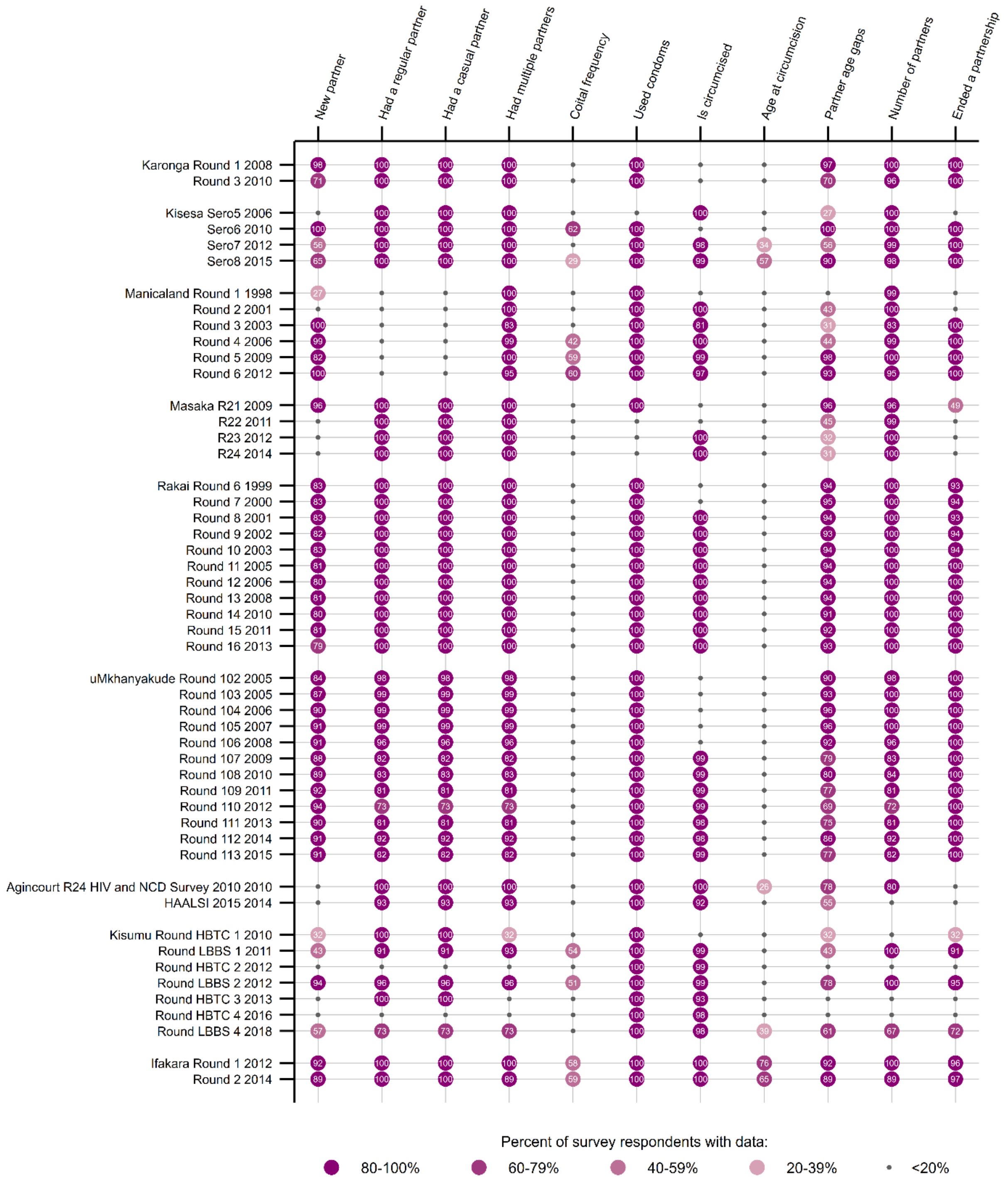

# Supplementary Methods: Additional information on methods

Description of studies

We use geographic names for each study because in several instances the data we use were collected over a period which spanned several incarnations of research project and institutional identities, with the geographical location being the constant. Eligibility for the individual studies was defined by residency within a contiguous area corresponding to a local administrative area, except in Manicaland where study areas were geographically defined but not contiguous. Manicaland, Masaka, Rakai and uMkhanyakude have changed their geographic size over time and we have used only their continuously surveyed areas.

Descriptive statistics were calculated as follows. For categorical variables we calculated the person-time and failures in each category of the variable, by study, sex and age group, for each imputation. We then took the mean of these estimates across all imputations and calculated the proportion of person time and failures in each category. For continuous variables (rates and prevalence of untreated infection), we calculated the estimate, by study, sex and age group, for each imputation and then took the mean across imputations.

For the construction of the time-varying variables, we needed to map the information from the cross-sectional surveys to the longitudinal demographic data. Some questionnaire responses included the date or age at which the status changed (for example, date of birth, age at circumcision) and when this was available we used it to split the data at that point and allocate the status to each portion of follow-up time accordingly. Some of the information was collected as the status at the time of the survey. This meant the timing of a change in status had to be inferred from the dates of the two reports which indicated there had been a change. For education we assumed the status change took place at the midpoint between the two reports because for young people, where status is most likely to change, this information was often collected more frequently, in the demographic data collection, than information collected in the individual surveys. Marital status was changed on the date of the new report, rather than the midpoint, because we initially we separately categorised the time at risk in the intervals between two different states (e.g. interval between single to married) as we expected these transition periods to be particularly high risk. However the numbers were too small to analyse these. The information about the numbers and types of partnerships, and behaviour with those partners, was assigned to the one year before each survey.

For variables where repeated measurement could produce some impossible combinations over time (such as going from “married” to “never married” or from having secondary education to only primary) we applied an algorithm to deduce the most likely status over time, which was based on the numbers and sequence of reports of the different status. This captured and removed anomalies which were likely the result of isolated mistakes in data capture and assigned any that still did not make sense as “unknown”.

# Supplementary Results

## Flowcharts showing participation in incidence cohort

Each study covers the entire population of a defined geographical area and the study participants are not therefore a probability sample but more akin to a census.  At the household level, participation in demographic surveillance is high.  The incidence cohort is not an actual cohort but something we construct for analysis based on data supplied as part of the ongoing studies.  Inclusion in the incidence cohort requires individual participation, by initially HIV negative individuals, in at least two survey rounds with study testing. This requires the individuals to be resident and present at the time of two rounds and to consent to HIV testing on multiple occasions. Some people were not observed in the study for long enough to have been offered two tests, others tested positive on the first occasion and these people were not eligible for inclusion in the incidence cohort.  Among those who had been in the study for long enough, some were never tested, and others were tested only once.  A small number had test results that precluded inclusion such as testing negative after previously testing positive.  The flowcharts in Supplementary Figure 2  show the numbers included in the incidence cohort in each study, by sex.  Overall, 74% of people ever enumerated in the demographic surveillance were eligible for inclusion in the incidence cohort and we had sufficient data to include 84% of those eligible. Some variations by study were due to differences in length of time that HIV testing was offered, participation levels in the studies and population mobility.

Supplementary Figure 3: Flowcharts for women and men in each study showing the number of people included for analysis in the incidence cohort. Numbers in incidence cohort include everyone who was included in at least one imputation. People could contribute data to both age groups and may therefore have been counted twice in the bottom row.

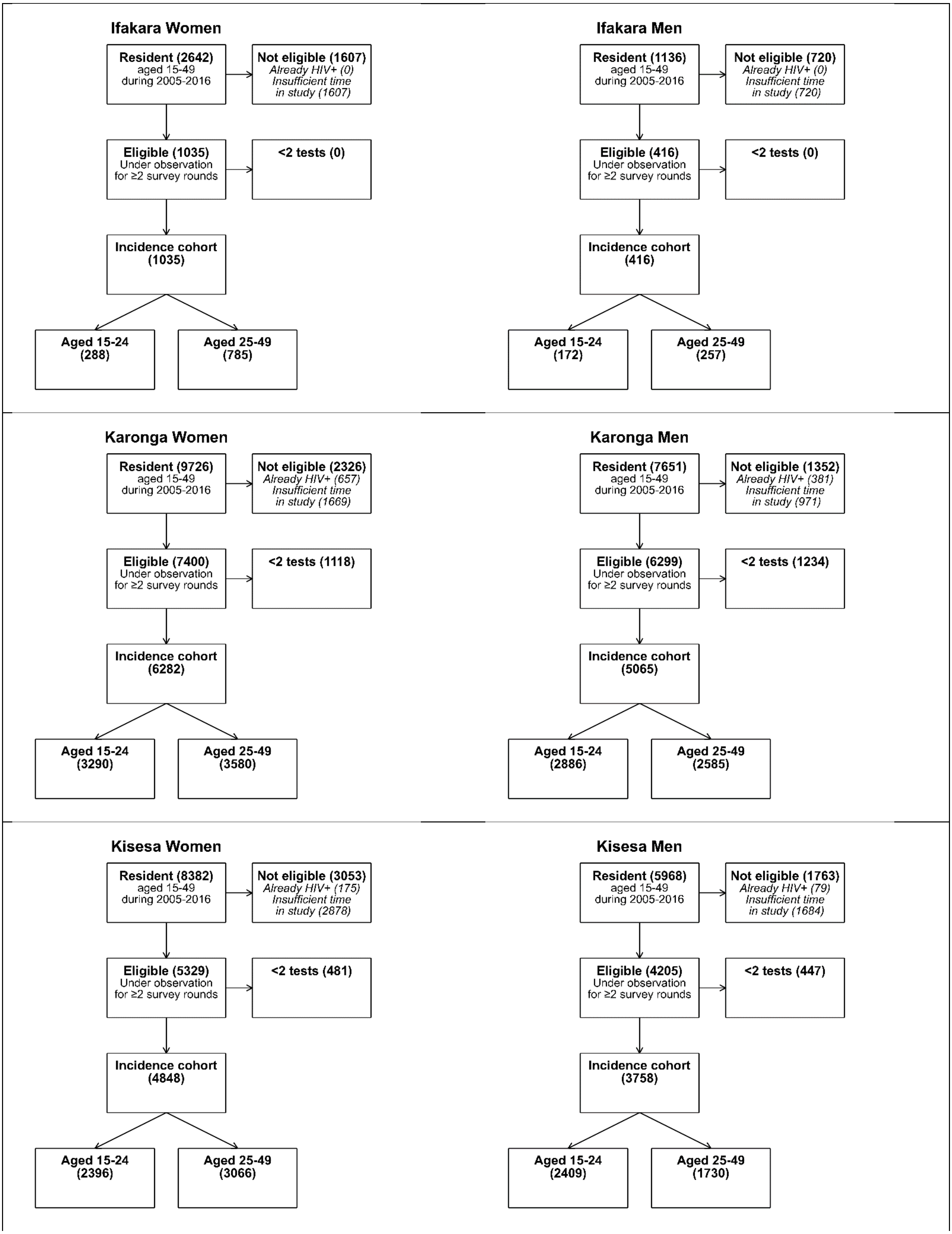

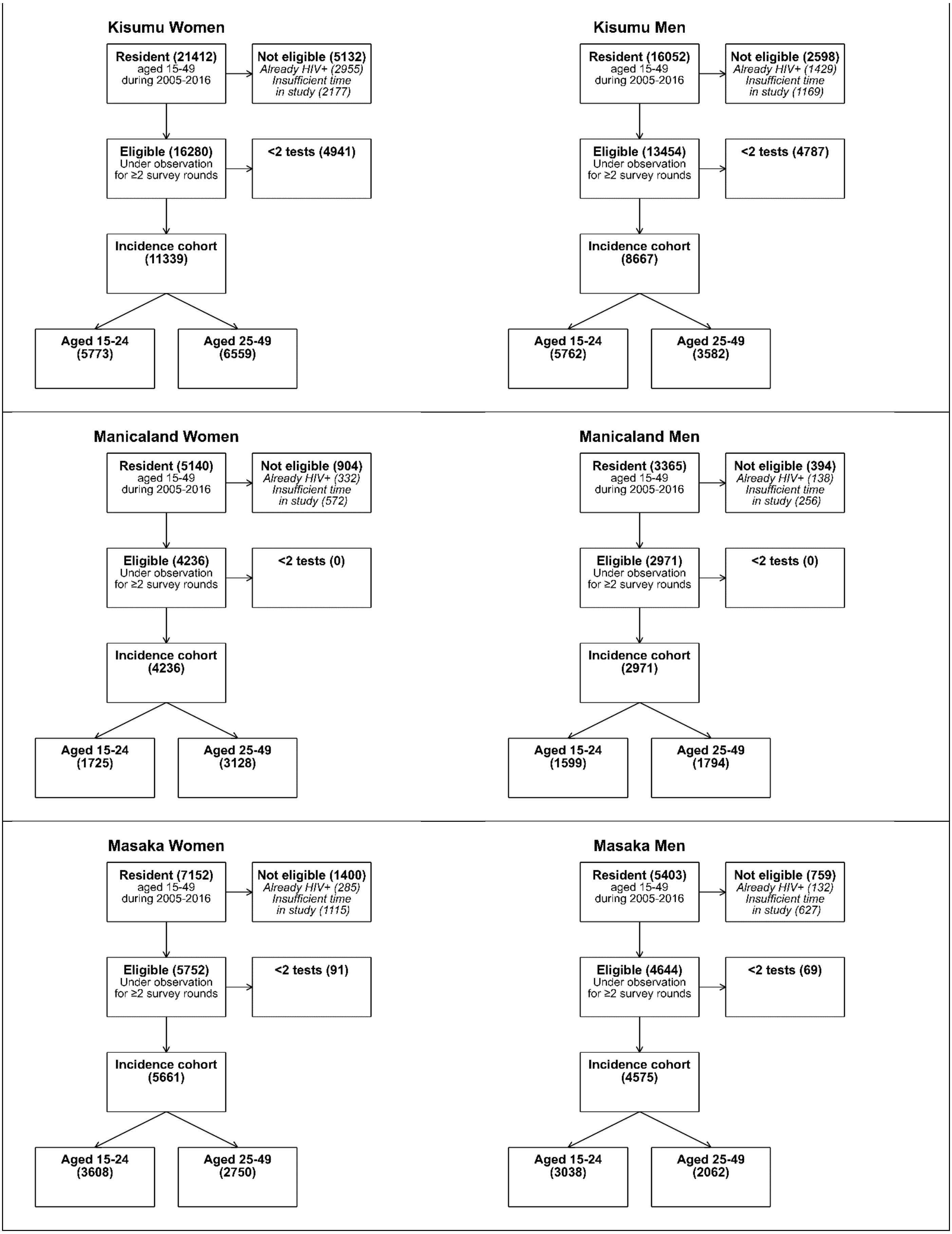
Kisumu Women
Resident (21412) aged 15-49 during 2005-2016
Not eligible (5132) Already HIV+ (2955) Insufficient time in study (2177)
Eligible (16280) Under observation for ≥2 survey rounds
<2 tests (4941)
Incidence cohort (11339)
Aged 15-24 (5773)
Aged 25-49 (6559)
Kisumu Men
Resident (16052) aged 15-49 during 2005-2016
Not eligible (2598) Already HIV+ (1429) Insufficient time in study (1169)
Eligible (13454) Under observation for ≥2 survey rounds
<2 tests (4787)
Incidence cohort (8667)
Aged 15-24 (5762)
Aged 25-49 (3582)
Manicaland Women
Resident (5140) aged 15-49 during 2005-2016
Not eligible (904) Already HIV+ (332) Insufficient time in study (572)
Eligible (4236) Under observation for ≥2 survey rounds
<2 tests (0)
Incidence cohort (4236)
Aged 15-24 (1725)
Aged 25-49 (3128)
Manicaland Men
Resident (3365) aged 15-49 during 2005-2016
Not eligible (394) Already HIV+ (138) Insufficient time in study (256)
Eligible (2971) Under observation for ≥2 survey rounds
<2 tests (0)
Incidence cohort (2971)
Aged 15-24 (1599)
Aged 25-49 (1794)
Masaka Women
Resident (7152) aged 15-49 during 2005-2016
Not eligible (1400) Already HIV+ (285) Insufficient time in study (1115)
Eligible (5752) Under observation for ≥2 survey rounds
<2 tests (91)
Incidence cohort (5661)
Aged 15-24 (3608)
Aged 25-49 (2750)
Masaka Men
Resident (5403) aged 15-49 during 2005-2016
Not eligible (759) Already HIV+ (132) Insufficient time in study (627)
Eligible (4644) Under observation for ≥2 survey rounds
<2 tests (69)
Incidence cohort (4575)
Aged 15-24 (3038)
Aged 25-49 (2062)

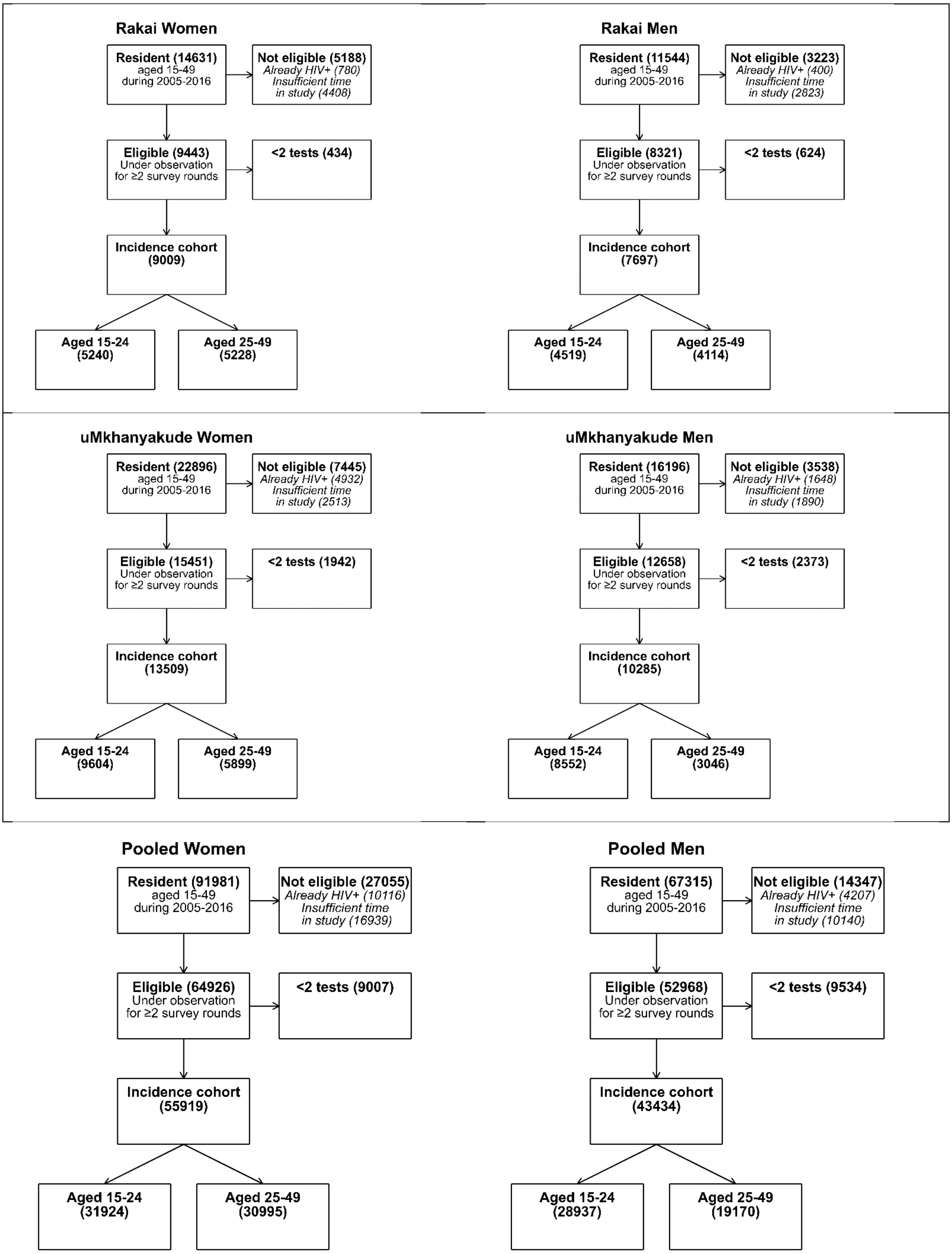
Rakai Women
Resident (14631) aged 15-49 during 2005-2016
Not eligible (5188) Already HIV+ (780) Insufficient time in study (4408)
Eligible (9443) Under observation for ≥2 survey rounds
<2 tests (434)
Incidence cohort (9009)
Aged 15-24 (5240)
Aged 25-49 (5228)
Rakai Men
Resident (11544) aged 15-49 during 2005-2016
Not eligible (3223) Already HIV+ (400) Insufficient time in study (2823)
Eligible (8321) Under observation for ≥2 survey rounds
<2 tests (624)
Incidence cohort (7697)
Aged 15-24 (4519)
Aged 25-49 (4114)
uMkhanyakude Women
Resident (22896) aged 15-49 during 2005-2016
Not eligible (7445) Already HIV+ (4932) Insufficient time in study (2513)
Eligible (15451) Under observation for ≥2 survey rounds
<2 tests (1942)
Incidence cohort (13509)
Aged 15-24 (9604)
Aged 25-49 (5899)
uMkhanyakude Men
Resident (16196) aged 15-49 during 2005-2016
Not eligible (3538) Already HIV+ (1648) Insufficient time in study (1890)
Eligible (12658) Under observation for ≥2 survey rounds
<2 tests (2373)
Incidence cohort (10285)
Aged 15-24 (8552)
Aged 25-49 (3046)
Pooled Women
Resident (91981) aged 15-49 during 2005-2016
Not eligible (27055) Already HIV+ (10116) Insufficient time in study (16939)
Eligible (64926) Under observation for ≥2 survey rounds
<2 tests (9007)
Incidence cohort (55919)
Aged 15-24 (31924)
Aged 25-49 (30995)
Pooled Men
Resident (67315) aged 15-49 during 2005-2016
Not eligible (14347) Already HIV+ (4207) Insufficient time in study (10140)
Eligible (52968) Under observation for ≥2 survey rounds
<2 tests (9534)
Incidence cohort (43434)
Aged 15-24 (28937)
Aged 25-49 (19170)

## Distribution of each risk factor by age, sex and study

Supplementary Data 1 and Supplementary Data 2 show, for men and women respectively, the distribution of person years in each study by socio-demographic characteristics and sexual behaviour, as a percentage of the total time spent in each age group. In these estimates we have excluded person time that could be not be classified due to a lack of information.

## Summary of results for each risk factor

**Calendar year**

The follow-up time was divided into three periods: 2005-2008, 2009-2012 and 2013-2016. The middle period included 40% of the time under observation with 30% in the earlier and later periods (Table 2). This varied somewhat by study due to differences in HIV data collection timing: there was no data in the last period for Karonga and Manicaland, and no data from the first period for Ifakara and Kisumu. The crude analysis showed incidence was lower in 2013-16 compared to 2005-08 in both age groups for men and women with crude HR ranging from 0.60 to 0.70. In the adjusted analysis which included calendar year, this association was seen for young women, older men and older women everywhere except uMkhanyakude and there was no evidence of confounding by other factors.

**Residence**

The classifications of residence type varied between studies but the majority of respondents lived in rural areas, or in small urban or peri-urban areas within a predominantly rural setting. Around a quarter of participants moved house during the period of observation. The 12 month period following a move accounted for less than 10% of the follow up time, and was highest for young women at 8%. For both sexes and both age groups there were associations with type of place of residence but, because these data weren't harmonised, it is not possible to find a generalisable pattern. In the crude analysis of pooled data (Table 3), residential mobility was associated with increased risk for younger women (HR 1.29), older women (HR 1.84) and younger men (HR 1.68). In the adjusted analysis this effect was only apparent for older women (HR of 1.592 (95% CI 1.268-1.998)).

**Education**

Most people had had primary education, accounting for approximately 60% of person time (Table 1). Secondary education was most common among young men and young women, and varied by study with uMkhanyakude (South Africa) and Manicaland (Zimbabwe) participants reporting higher levels of education. The vast majority of people in Manicaland had secondary education but very few had tertiary education whereas in uMkhanyakude this accounted for 22% of older men's person time and 17% of older women's (Supplementary Data 1 and 2). In the crude analysis secondary education was protective for young women and a risk for older women. After adjusting for the other factors, secondary education was protective only for young women (adj HR 0.70 95% CI 0.56-0.87). There were no associations with education for men.

**Marital status**

Marital status varied by age and sex, with 91% of younger men's time prior to marriage/cohabitation, compared to only 72% of young women's time whilst around 60% of older men and women's time was spent married or cohabiting with a partner (Table 2). Marital status differed in uMkhanyakude compared to the other studies; for older men 81% of person time was in the never married category, whereas elsewhere currently married was the most common (63% to 76% of person time) (Supplementary Data 1). For women, the same pattern was observed: amongst young women in the other studies between 14% and 44% of person time was classified as currently married compared with 0% in uMkhanyakude. Among older women in uMkhanyakude 58% of person time was spent unmarried and only 2% married, whereas elsewhere between 62% and 90% of person time was spent currently married (Supplementary Data 2).

The effects of marital status on HIV incidence differed by sex and age and were similar in the crude and adjusted analysis. For young women, older women and older men, being formerly married was associated with increased risk

of HIV acquisition, with the highest HR for young women (2.748 95% CI 1.654-4.566). This association was not seen for young men but there were only 249 men who fell into this category during the study period. Amongst young men, being never married (the most common status) was associated with a much lower risk of HIV acquisition compared to being currently married (adj HR 0.378 95% CI 0.233-0.612). For older women, never having been married was a risk (adj HR 2.220 95% CI 1.716-2.874).

**Completeness of sexual behaviour data**

Data on sexual partners and recent behaviour was available for almost 80,000 person years. However coverage was incomplete, mainly because data were not sought for all periods in all studies, and around 90% of respondents contributed some time to the unknown category for these variables. The amount of person time for which there was no data was lower for younger people (73% for men and 71% for women) than for older people (82% for men and 80% for women).

**Number of partners**

Considering the person time for which there was data on sexual behaviour, there were sex and age differences in the partnerships reported. Most of men's, and the vast majority of women's classified person time was classed as having either one or zero partners. There was a large sex difference, with 10% of young men's time and 26% of older men's time spent in a period where they had more than one partner compared to 1% and 2% for younger and older women respectively (Table 2).

Most of young men's time (60%) was during a period where they had not had a partner in the past 12 months, this figure was 47% for young women and just 10% and 15% for older men and women respectively. We investigated the association between numbers of sexual partners in the previous year, and HIV incidence and the effects of having one or no partner compare to more than one. Although having more than one partner was associated with HIV incidence in the crude analysis for women and young men, and having two partners compared to one was associated with incidence for young women, there were no associations observed in the adjusted models.

**Types of partners**

With regard to non-spousal, non-cohabiting partners, the amount of time spent in a partnership with a regular partner was similar by age and sex, ranging from 22% to 30% in the pooled data (Table 2 and Supplementary Data 1 and 2). The amount of time spent in a partnership with a casual partner was lower and similar for women and young men (10-12%) but higher for older men (21%). There were some differences between the studies in the prevalence on non-spousal, non-cohabiting partnerships, and also some differences in ascertainment of these partners; this information was not available for people in Manicaland and, in Masaka, Karonga and Kisumu, this information was not collected in every survey round.

The types of sexual partner were associated with HIV risk. When compared to people with all other types (including no partner) the HR for having a casual partner were above 1 in all groups but significant only for young women, with an adjusted HR of 1.83 (95% CI 1.39-2.41).

Regular partners were also a risk in all groups in the crude analysis and for women and older men in the adjusted analysis, with the adjusted HR for older men showing the strongest effect (1.83 (95% CI 1.24-2.68)). In the adjusted analysis, no measures of partner type or number were associated with HIV incidence for young men.

**Age differences between partners**

In the pooled data, young men's time and half of young women's time in partnerships were with partners their own age. For young women for the other half of the time they were in partnerships it was with older partners, most of which were within 5-9 years of their own age. This distribution was similar for older women except in Kisesa and Ifakara, Manicaland and in Kisumu where are larger proportion of time was spent with partners who were older, and a larger share who were more than 10 years older. For older men, slightly more time was spent in partnerships with partners who were 5 or more years younger than them. In Kisesa, Manicaland, Masaka, Rakai and Ifakara the

majority of men's time was spent in partnerships where the age gap was more than 5 years. Age differences between partners were not associated with HIV incidence.

**New partnerships**

For men, having a new partner was a fairly common experience with around a third of male respondents reporting a new partner in at least one survey. Fewer women reported new partners, with just under a third of young female respondents and only a tenth of older female respondents reporting having acquired a new partner. The amount of person time spent in a new partnership (within the first 12 months) was similarly varied with around a fifth of men's time and 11% of young women's and 5% of older women's time spent with new partners. There was some variation by study, notwithstanding some extreme and somewhat implausible results for Ifakara. Older women ranged from 3.7% of time in Karonga to 7.7% in Kisesa. younger women ranged from 8.0% in Kisumu to 12.1% in Kisesa. Older men ranged from 13.0% in uMkhanyakude to 28.7% in Kisesa. Older men ranged from 13.4% in Kisumu to 29.4% in Karonga.

In the crude analysis, new partnerships were associated with increased risk in all groups. In the adjusted models, the association remained for women, with the risk greater for older women than for young women (adj HRs comparing new partnerships versus none 1.96 and 1.36 respectively).

**Coital frequency**

Coital frequency was not collected in all studies, and not in every round in any study. Where data were available, most of respondents' time was in periods where they reported sex more than once a week, taking into account all of their partners, and ranging from 51% for young men, 63% for young women, 70% for older women to 76% for older men. Coital frequency was not associated with HIV incidence.

**Male circumcision**

Data on male circumcision was collected everywhere except Karonga. Men were asked if they were circumcised but no attempt was made to distinguish traditional from medical male circumcision. Most men were asked if they had been circumcised at the time of the survey, rather than the age at which they were circumcised. For those men, their circumcision status is unknown prior to their first report which amounts to 69% of person-time for young men and 71% for older men.

Among those for whom circumcision status is known, the proportion of young men's person time that was known to be post-circumcision ranged from 6% in Masaka to 92% in Ifakara. There was a large difference in the nearby studies in Uganda, Masaka and Rakai (52%). Rakai hosted one of the trials on the effectiveness of male circumcision for HIV prevention. In total, 14% of the time contributed by young men for this analysis was known to be post-circumcision, 45% of the time where status was known. The picture was similar for older men, where 18% of men's time was known to be post-circumcision, representing 63% of the time for which status was known.

Circumcision was strongly protective for HIV incidence but, in these settings, this was only apparent for young men, with an adjusted HR of 0.38 (95% CI 0.21-0.70).

**Condom use**

Participants were asked about condom use with all their partners in the last year. Very few reported consistent condom use with all partners so only a small proportion of time fell in that category (younger men 3.2%, older men 1.9%, younger women 2.0%, older women 1.5%). There was a big difference by age with 57% of older men's time classified as 'no condom use' compared to 18% of younger men's time, and, for women, 60% for older women compared to 25% for younger. Looking only at person-time spent in partnerships there are big differences by age: 9% of young men's time is spent as a consistent user versus 2% for older men and for women this is 5% versus 2% (Supplementary Data 3 and 4). Only 48% of young men's time is classed as never using compared to 74% of older men's, 56% of younger women's compared to 82% for older women. Most condom users report inconsistent use (use with some partners but not others, or inconsistent use within a partnership) and there are differences between

studies in use with the lowest use in Kisumu for both men and women in both age groups. Use is highest in Ifakara, Karonga and uMkhanyakude for both age groups and both sexes.

In the crude analysis, inconsistent condom use was a marker of risk with older men and older women having higher HIV incidence than non users (HR 1.92 and 2.59 respectively). In the adjusted models this association was still apparent for older women with those reporting inconsistent use having twice the incidence hazard.

**Ending a partnership**

Ending a partnership was measured as having had a partnership in the last year which was not ongoing at the time of the survey. This information is available for around 40% of respondents. A partnership ending was less common for women than men. Older men had slightly more time classed as 'partnership ended' than younger men (16% versus 12%) but the difference was smaller for women (5% versus 6%). There were variations by study with Kisumu consistently the lowest. For young men and women, Rakai had the highest proportion of person time around the end of a partnership (28% and 10%) and for older men that was Ifakara (28%) and for older women Kisesa (11%). Ending a partnership was associated with increased risk only among young women but this effect was not seen in the adjusted model.

**Community partner acquisition and loss rates**

Partner acquisition rates, available only for Karonga, Kisesa and Rakai, were highest for young men, and higher for men than for women. Partner loss rates were lower than acquisition rates, except for older men (Table 2).

In the crude analysis partner acquisition rates among peers were associated with higher incidence for men and older women (Table 3) but, after controlling for their own behaviour and characteristics and the prevalence of untreated HIV infection in potential opposite sex partners the association remained only for older men and older women (Table 4) The effect of a one point increase in the men's partner acquisition rate was a 3% increase in the HIV incidence hazard for older women and for older men (adjusted HRs 1.03, 95% CI 1.01-1.05 and 1.03, 95% CI 1.01-1.04, respectively).

**Prevalence of untreated HIV infection among potential partners of the opposite sex**

The prevalence of untreated HIV infection in potential opposite sex partners was very different for men and women, varied by age and between studies.

The prevalence of untreated HIV infection in potential sexual partners was associated with HIV incidence for men and women in both age groups (Table 3). The effect was strongest for young men where a one percentage point increase in this prevalence was associated with a 7% increase in the hazards of HIV acquisition (Adjusted HR 1.07. 95% CI 1.05-1.09). This association was not apparent in the models which included age and calendar time (Supplementary Data 7 to 10). The prevalence of untreated infection is correlated both with age and with calendar time and, when included in the same models, these variables more efficiently explain the variation in the outcome. However, we opted to retain the prevalence of untreated infection since it is the more proximate determinant.

**Study level effect modification**

The HR differed by study for education (women and older men), mobility and new partnerships (young men and women), and untreated prevalence (all). Pooled models including interaction terms for effect modification by study were fitted but the data were generally too sparse to permit estimation of these terms for the categorical variables. For the prevalence of untreated infection there was some evidence of effect modification by study.

Supplementary Data 1: Distribution of person years by socio-demographic characteristics and sexual behaviour among the men in each study for whom there is information, as a percentage of the total time spent in each age group.

This table is in the Supplementary Data Excel file

Supplementary Data 2: Distribution of person years by socio-demographic characteristics and sexual behaviour among the women in each study for whom there is information, as a percentage of the total time spent in each age group.

This table is in the Supplementary Data Excel file

## Crude HR for HIV incidence for all risk factors in each study, for men and women by age

The crude HR was estimated for each risk factor separately for men aged 15-24, men aged 25-49, women aged 15-24 and women aged 25-49 and these results are shown in Supplementary Data 3 for young women, Supplementary Data 4 for older women, Supplementary Data 5 for young men and Supplementary Data 6 for older men. Each table gives the crude HR and 95% CI and the results where the CI do not include 1 have been shaded to indicate a risk (red) or protective association (green). In some instances it was not possible to estimate the HR either because there were no seroconversions observed (indicated with **) or because there were no person years observed in that category (-) or because the model could not be fitted (*).

**Education**

A protective effect of secondary compared to primary education was seen for young women in Kisesa (0.17, 0.03-0.89) and Rakai (0.69 0.46-1.03) with consistent HR elsewhere except Karonga (Supplementary Data 3). Among older women the pattern was different, with secondary education a risk in Manicaland (2.01 1.29-3.12), and HR above 1 although with wide confidence intervals elsewhere, except Karonga (Supplementary Data 4). Tertiary education was protective in Rakai only (0.40 0.19-0.84) . For young men, although the HR were below 1 everywhere except Manicaland, the confidence intervals for a protective effect of secondary compared to primary education included 1 giving a pooled estimate of 0.71 (0.48-1.03) (Supplementary Data 5). There was no pattern for older men with three studies showing a protective effect of secondary education and three showing an increased risk, all with confidence intervals that included 1 except for Rakai where both secondary and tertiary education were associated with lower hazards (Supplementary Data 6).

**Residence**

It was not possible to harmonise residence classifications and so the estimates are not directly comparable between the studies. Participants in Ifakara, Kisumu, Masaka and Rakai all lived in the same type of area so no comparison was possible.

There were no widespread associations between residence and hazard of HIV infection for women or men. Young women living in peri-urban areas in Kisesa and uMkhanyakude had a higher hazard of HIV infection than counterparts in rural areas (2.41, (1.11-5.23) and 1.23 (1.10-1.38) respectively). In Kisesa this risk was higher than for urban areas but in uMkhanyakude the HR for urban and peri-urban areas were similar (Supplementary Data 3). The pattern was similar for older women (Supplementary Data 4) and for men (Supplementary Data 5 and 6) where the only significant association were seen in uMkhanyakude. For older men there was a borderline protective effect in Manicaland for those living in roadside and subsistence farming locations compared to agricultural estate residents (Supplementary Data 6).

**Mobility**

Residential mobility was commonly a risk for HIV though most of the CI for individual study estimates included 1. For young women, it was a risk everywhere except Manicaland, with a pooled HR of 1.29 (1.04-1.60). For older women it was a risk everywhere with a pooled HR of 1.84 (1.47-2.31). For young men it was a risk everywhere except Kisumu, though the HR could not be estimated in Ifakara and Kisesa, and the pooled HR was 1.68 (1.11-2.55). Among

older men, the HR were all above 1 (except Ifakara where no estimate was possible) but the CI for the pooled HR (1.30) includes 1 (0.94-1.81).

**Calendar Time**

There was a decrease in HIV incidence over time in all studies for men and women in both age groups and this is borne out in the estimated HR which are below 1 for 2013-16 compared to the earlier period in all comparison except for older women in uMkhanyakude where the hazard had increased compared to 2005-9 (1.28, 1.03-1.58). The pooled estimates for the size of the decline were similar for men (0.67 (0.50-0.89) and 0.64 (0.51-0.80) for younger and older respectively) and women (0.70 (0.61-0.81) for younger women). A pooled HR was not useful for uMkhanyakude given the strong effect modification by study (Supplementary Data 4).

**Population-level measures of partnership dynamics** could be estimated in three studies: Karonga, Kisesa and Rakai. Rates of acquisition and loss were calculated for each individual's contemporaries by age, sex, calendar period and study and for individuals of the opposite sex in the age range of potential partners. In most of the comparisons, the CI for the HR for one unit increase in the rate (acquisitions or losses/100 person years) were too wide to show a clear and consistent pattern of association. Among young women, the HR for loss rates among peers and potential partners are all above 1 (Supplementary Data 3) whereas for older women acquisition rates appear more consistently associated with risk, significantly so for Karonga (1.05, 1.00-1.10) and Rakai (1.02, 1.01-1.04) and with a pooled HR of 1.04 (1.00-1.09) (Supplementary Data 4). The acquisition rate among peers was associated with higher hazards of HIV seroconversion for young men (pooled HR 1.01 (1.00-1.03) Supplementary Data 5). For older men, the acquisition rate amongst peers was positively associated with HIV seroconversion (pooled HR 1.03 (1.01-1.04) Supplementary Data 6) and the acquisition rate among partners was associated more strongly (pooled HR 1.08 (1.03-1.14)). The partner loss rate among peers was only associated in Rakai (1.03 (1.01-1.05)). The loss rate among the opposite sex was associated with HIV seroconversion (pooled HR 1.15 (1.01-1.30) Supplementary Data 6) but the loss rate among peers was not.

**Current Marital Status**

For women and older men, the risk among formerly married people was higher than among those currently married. This association was significant for younger women in Rakai (Supplementary Data 3), for older women in Kisumu, Manicaland, Masaka and Rakai (Supplementary Data 4) and for older men in Manicaland (Supplementary Data 6). For younger men, few were formerly married so estimates were possible only in Karonga and Rakai where the HR were above 1 but the CI included 1. However in this age group there was a protective effect of being never married, with all HR below 1 and a pooled HR of 0.32 (0.20-0.51)(Supplementary Data 5). This was not seen for young women (Supplementary Data 3) and being never married was a risk among older men (2.67, 2.26-3.15, Supplementary Data 6) and older women (pooled HR 2.5, 1.96-3.22, Supplementary Data 4)

**Number of partners in the last year**

For women, the HR for two or more partners in the year before the survey could not be estimated because there weren't enough failures observed among women who had 2 or more partners except in Rakai (both age groups) and uMkhanyakude (young women only) where it was associated with an increased risk compared to women who had only 1 partner. Having no partners was protective for young women (Pooled HR 0.51, 0.43-0.60, Supplementary Data 3) but for older women this effect was only observed in uMkhanyakude (0.53, 0.29-0.94, Supplementary Data 4). Estimates for young men were also hampered by small numbers of events. In Masaka, Rakai and uMkhanyakude there was a upwards trend in the HR with increasing numbers of partners but this was not significant (Supplementary Data 5). Elsewhere estimates were not possible. For older men, HR were estimable for at least one category in all studies and increasing trends were observed. The HR comparing 3 or more partners to 1 partner were significant for men in Karonga and Rakai only (Supplementary Data 6).

**More than one partner in the last year**

This measure also suffered from small numbers of events and could be estimated for Rakai and uMkhanyakude only for young women, Masaka only for older women, Masaka, Rakai and uMkhanyakude for young men and everywhere

except Kisumu for older men. The estimates for women all showed a significant risk (Supplementary Data 3 and 4) with pooled HR of 3.03 (1.83-5.02) for younger women and 3.15 (1.77-5.60) for older women.

Among younger men, there was a positive association with HIV seroconversion with a pooled HR 2.32 (1.46-3.70) Supplementary Data 5). For older men the association was significant in Karonga and Rakai and in the same direction in all studies but the pooled HR CI included 1 (1.32, 0.94-1.86, Supplementary Data 6).

**Casual and regular partnerships**

There was not enough seroconversions observed in most studies to estimate the effects of having a casual and/or regular partner on the hazard of seroconversion. Where this could be estimated, having either partner increased the hazards of acquiring HIV. For young women, the pooled HR for a casual partnership was 1.57 (1.22-2.02) and 1.75 (1.48-2.08) for a regular partnership. For older women, the pooled HR was 1.27 (0.86-1.88) for a casual partnership and 2.34 (1.82-3.01) for a regular partnership. For young men, the pooled HR for casual partnerships was 1.42 (0.91-2.22) and for regular partnerships 1.55 (1.09-2.22). For older men, the HR for casual partnerships was 1.38 (0.91-2.11) and the HR for regular partnerships was 3.17 (2.18-4.59).

**Age differences**

For young women, being in a partnership with a partner who was 10 or more years older compared with having a partner within 5 years of their own age showed a positive association with HIV seroconversion with wide confidence intervals (Pooled HR 1.39 (0.90-2.14) Supplementary Data 3) in all studies except Rakai. A similar, but smaller effect was observed for partners who were 5-10 years older, except in Ifakara, but again this could be due to chance (1.08 (0.84-1.40) Supplementary Data 3). This pattern was not apparent for older women, in fact HRs were below 1 for women with older partners, except in Kisesa, with pooled HR of 0.81 (0.58-1.11) for partners 5-10 years and 0.65 (0.41-1.01) for those 10+ years older (Supplementary Data 4).

Among young men, there weren't enough seroconversions to be able to estimate the study-specific effects of age disparate partnerships, except for young men in uMkhanyakude (Supplementary Data 5). The pooled HR for partnerships where the man was 5-10 years older was 1.29 (0.44-3.79). Almost no men in this age group had partners who were 10 or more years younger. Among older men, there was no pattern, and no significant associations (Supplementary Data 6).

**Prevalence of untreated infection**

In the pooled data, the prevalence of untreated HIV infection in potential partners of the opposite sex was associated with HIV seroconversion for men and women in both age groups. The increase in HIV seroconversion hazard for one percentage point increase in the prevalence of untreated infection was lowest for young women (pooled HR 1.02 (1.01-1.03) Supplementary Data 3) and highest for young men (pooled HR 1.07 (1.05-1.09) Supplementary Data 5). The estimates for older women (pooled HR 1.05 (1.03-1.06) Supplementary Data 4) and older men (pooled HR 1.06 (1.05-1.07) Supplementary Data 6) were very similar. These pooled estimates belied some study level differences. Among young women the HR was as high as 1.09 (1.02-1.16) in Manicaland but below 1 (0.95 (0.60-1.49)) in Ifakara. The pattern was more mixed for older women with estimates from Kisesa, Masaka and Ifakara below 1 (HR 0.95, 0.97 and 0.96 respectively). The HR for young men were the most varied, ranging from 0.67 (0.11-3.97) in Ifakara to 1.43 (1.03-1.97) in Rakai and 1.21(1.09-1.34) in uMkhanyakude. Among older men, only in Ifakara was the HR below 1 (0.89 (0.49-1.63)) but it was very close to 1 (HR 1.01) in Kisesa, Manicaland, Masaka and uMkhanyakude.

**New partners**

Having acquired a new partner in the year before the survey was associated with increased HIV seroconversion hazard for young women everywhere except Ifakara (pooled HR 1.80 (1.44-2.24) Supplementary Data 3), for older women in all studies where this could be estimated (pooled HR 2.52 (1.75-3.64) Supplementary Data 4), for young

men in Masaka and uMkhanyakude, giving a pooled HR of 1.49 (1.03-2.15) (Supplementary Data 5) and for older men in Karonga, Rakai and uMkhanyakude (pooled HR 1.48 (1.00-2.19) Supplementary Data 6).

**Coital frequency**

Data on coital frequency was available in some surveys from Ifakara, Kisesa, Kisumu and Manicaland. Compared to those who had sex less than once a week there was no difference in the hazards of HIV seroconversion among people who had more frequent sex.

**Condom use**

Consistent and inconsistent condom use across all partnerships was compared to non-use. For young women, few events among condom users meant that this could only be estimated in Rakai and uMkhanyakude (Supplementary Data 3). In both studies condom users had a higher risk of HIV seroconversion than non-users but this could be due to change. For older women (Supplementary Data 4), estimates were possible for Karonga and Rakai and, whilst Rakai showed the same increased risk as before, which was significant for inconsistent users, the direction of the effect was protective in Karonga but the confidence intervals were very wide. For young men, estimates were possible in Masaka, Rakai and uMkhanyakude and these results were very mixed with no pattern of association (Supplementary Data 5). Amongst older men, inconsistent condom use was positively associated with HIV incidence (pooled HR 1.92 (1.36-2.70) Supplementary Data 6)

**Ended partnership**

Having ended a partnership in the year before the survey was associated with increased risk of HIV seroconversion for young women (pooled HR 1.64 (1.15-2.35) Supplementary Data 3) in all studies where this could be estimated (there were too few failures to make an estimate in Ifakara, Kisesa, Kisumu and Manicaland). Among older women an association was only observed in Rakai (HR 3.26 (1.28-8.27) Supplementary Data 4) and there were no associations observed for men in any study.

**Circumcision**

Circumcision was strongly protective for young men (pooled HR 0.36 (0.20-0.66) Supplementary Data 5) everywhere except Kisesa (2.30 (0.25-21.24). Among older men the HR were below 1 again, except in Kisesa, but the confidence intervals around the pooled HR were wide (pooled HR 0.88 (0.61-1.27) Supplementary Data 6).

Supplementary Data 4: Crude HR for HIV incidence in each study among women aged 15-24

This table is in the Supplementary Data Excel file

Supplementary Data 5: Crude HR for HIV incidence in each study among women aged 25-49

This table is in the Supplementary Data Excel file

Supplementary Data 6: Crude HR for HIV incidence in each study among men aged 15-24

This table is in the Supplementary Data Excel file

Supplementary Data 7:  Crude HR for HIV incidence in each study among men aged 25-49

This table is in the Supplementary Data Excel file

## Adjusted risk factor models for HIV incidence using all studies' pooled data

We fitted four variants of multivariate piecewise exponential models for each age and sex group and then a most parsimonious model based on the results of those four. The results of the four alternative models are shown in Supplementary Data 7, Supplementary Data 8, Supplementary Data 9, Supplementary Data 10. Each model included all the factors which had shown an association in the crude analysis. The first variant was a proportional hazards model with a fixed effect for study (i.e. a different baseline hazard in each study), the second additionally included an interaction term between study and age to allow study-specific variation in the shape of the hazard function by age and the third additionally included an interaction between study and the prevalence of untreated infection. The fourth variant included all the risk factors from the crude analysis but did not adjust for age or calendar year, on the grounds that these were not directly associated with HIV incidence but were proxies for factors that were more directly associated. Supplementary Data 7 – 10 also show the mean, averaged across imputations of the number of seroconversions, people and person-time included in the model.

For young women, and leaving aside age and calendar year, the same terms were significant in all four models: secondary education (HR ~0.7) , former marriage (HR ~2.7), regular partners (HR ~1.8) and casual partners (HR ~1.9). In the model that omitted age and calendar year, new partners (HR 1.8) and the prevalence of untreated infection in potential partners (HR ~1.01) were also important. The HR for education, marriage, regular and casual partners were similar in all models. Those for new partnerships and untreated prevalence were larger in the model without age and calendar year. In the crude analysis there was an interaction between having a new partner and having a regular partner but this was less evident in the adjusted model, with the observed effect attributed to having regular partners. The effects of age and study were modelled differently in the three models and the effect of age on incidence differed between the studies, making the models with the interaction preferred over the model with proportional hazards.

Among older women, in all four variants of the model, the following were important, and had similar HR: moving house, never being married, former marriage, new partners. In the model without age and sex the prevalence of untreated infection was significant. Marital status was slightly different in the fourth model compared to the other three; the effect of no marriage versus current marriage more pronounced and that of former marriage was smaller.

For young men, being never married and being circumcised were strongly protective in all models, with the HR for marriage around 0.5 and for circumcision of 0.37. In the model without age and sex, the prevalence of untreated infection was also important with an HR of 1.06 (95% CI 1.04-1.09). Calendar time, moving house, education, and the number, types and newness of partners were not important.

For older men, in all models, former marriage and regular partners were risks but no associations were observed for new or casual partners, education, moving house, circumcision or ending a partnership. In the fourth model, prevalence of untreated infection was also important.

Supplementary Data 7:  Adjusted HR for HIV incidence among women aged 15-24 from four multivariate models

This table is in the Supplementary Data Excel file.

Supplementary Data 8: Adjusted HR for HIV incidence among women aged 25-49 from four multivariate models

This table is in the Supplementary Data Excel file.

Supplementary Data 9:  Adjusted HR for HIV incidence among men aged 15-25 from four multivariate models

This table is in the Supplementary Data Excel file.

Supplementary Data 10: Adjusted HR for HIV incidence among men aged 25-49 from four multivariate models

This table is in the Supplementary Data Excel file.

## Sensitivity analysis

To assess the influence each study exerted on the pooled results we carried out a meta-analysis of the study-specific adjusted HR for each age and sex group and repeated this leaving out one study at a time. These results are shown in Supplementary Table 2.   The table shows the  range of HRs obtained when repeating the meta-analysis removing one study at a time, and the HR estimates by meta-analysis using all  studies. These are based on the  estimates obtained from the preferred multivariate model for each age and sex group, fitted to each study's data. They show that all the estimates are robust to the removal of a single study,  demonstrating that the findings are not driven by any one study.

## Models including measures of partnerships dynamics in the community

Supplementary Data 11 and Supplementary Data 12 give the adjusted HR for men and women from preferred multivariate models repeated for the three studies which had partnership dynamics data (Karonga, Kisesa and Rakai) and including these variables. The preferred model was fitted to the data from these three studies, and is also shown for comparison with the results from pooled data from all eight studies (given in Table 4).  Partnership dynamics was described by 1) the partner acquisition rate among people of similar age and sex as the respondent, in the same study, and during the same calendar period; 2) the partner acquisition rate among potential partners of the opposite sex; 3) the partner loss rate among contemporaries and 4) the partner loss rate among potential partners of the opposite sex.

The only measure of partnership dynamics associated with HIV incidence in the adjusted models was the partner acquisition rate amongst male peers, for older men, and amongst potential male partners for older women. No measures were associated with HIV incidence among young people.

The effect of regular and casual partnerships was more pronounced for older people in these studies, compared to the pooled data. For older men and older women the effect of regular partner was no longer significant once PAR among men was controlled for and, for older women, the effect of former marriage was increased.

Supplementary Table 2: Results from leave-one-out meta-analysis to assess the influence of individual studies. Lowest and highest HR obtained and the pooled HR estimated by meta-analysis of all study specific estimates.

| Risk factor | Young Women Lowest HR | Young Women Highest HR | Young Women Pooled HR | Older Women Lowest HR | Older Women Highest HR | Older Women Pooled HR | Young Men Lowest HR | Young Men Highest HR | Young Men Pooled HR | Older Men Lowest HR | Older Men Highest HR | Older Men Pooled HR |
|---|---|---|---|---|---|---|---|---|---|---|---|---|
| Secondary education | 0.75 (0.61-0.92) | 0.82 (0.64-1.07) | 0.77 (0.63-0.94) | | | | 0.59 (0.37-0.92) | 0.69 (0.46-1.04) | 0.64 (0.46-0.90) | | | |
| Never married | 1.05 (0.79-1.40) | 1.22 (0.86-1.72) | 1.14 (0.87-1.48) | 1.75 (1.27-2.43) | 2.13 (1.60-2.84) | 1.95 (1.51-2.51) | 0.41 (0.26-0.64) | 0.49 (0.25-0.95) | 0.42 (0.28-0.65) | 1.08 (0.81-1.44) | 1.20 (0.92-1.57) | 1.14 (0.90-1.44) |
| Formerly married | 2.76 (1.53-4.99) | 3.77 (2.30-6.17) | 3.28 (2.17-4.96) | 1.86 (1.56-2.21) | 2.00 (1.69-2.37) | 1.93 (1.64-2.26) | 1.50 (0.47-4.84) | 2.65 (0.26-27.33) | 1.68 (0.59-4.79) | 1.39 (1.07-1.81) | 1.74 (1.30-2.31) | 1.57 (1.22-2.02) |
| Regular partner | 1.75 (1.43-2.14) | 2.38 (1.37-4.13) | 1.81 (1.50-2.19) | | | | | | | 1.61 (0.93-2.81) | 2.06 (1.27-3.33) | 1.85 (1.29-2.66) |
| Casual partner | 1.83 (1.03-3.27) | 1.87 (1.40-2.48) | 1.86 (1.44-2.40) | | | | | | | | | |
| New partner | 1.28 (1.03-1.60) | 1.39 (0.76-2.54) | 1.29 (1.04-1.61) | 1.59 (1.03-2.46) | 2.01 (1.17-3.46) | 1.76 (1.23-2.53) | | | | | | |
| Condom use | | | | 1.91 (1.20-3.02) | 2.28 (1.70-3.07) | 2.15 (1.67-2.78) | | | | | | |
| Circumcision | | | | | | | 0.24 (0.11-0.52) | 0.29 (0.14-0.63) | 0.26 (0.13-0.53) | | | |
| Prevalence of untreated infection in opposite sex potential partners | 1.02 (1.00-1.03) | 1.03 (1.01-1.06) | 1.02 (1.00-1.04) | 1.02 (1.01-1.04) | 1.03 (1.02-1.04) | 1.03 (1.02-1.04) | 1.06 (1.02-1.10) | 1.07 (1.05-1.09) | 1.06 (1.03-1.10) | 1.01 (0.99-1.02) | 1.02 (1.00-1.04) | 1.01 (1.00-1.03) |

Supplementary Data 11: Adjusted HR for selected risk factors for women in Karonga, Kisesa and Rakai including measures of partnership dynamics, by age for 2005-16 inclusive

This table is in the Supplementary Data Excel file.

Supplementary Data 12: Adjusted HR for selected risk factors for men in Karonga, Kisesa and Rakai including measures of partnership dynamics, by age for 2005-16 inclusive

This table is in the Supplementary Data Excel file.

Supplementary Data 13: Adjusted HR for selected risk factors for 2013-16 only, based on data from Kisesa, Masaka, Rakai, uMkhanyakude, Kisumu and Ifakara.

This table is in the Supplementary Data Excel file.

# Supplementary References


1. Lopman B, Nyamukapa C, Mushati P, et al. HIV incidence in 3 years of follow-up of a Zimbabwe cohort--1998-2000 to 2001-03: contributions of proximate and underlying determinants to transmission. *Int J Epidemiol* 2008; **37**(1): 88-105.
2. Edelstein ZR, Santelli JS, Helleringer S, et al. Factors associated with incident HIV infection versus prevalent infection among youth in Rakai, Uganda. *Journal of epidemiology and global health* 2015; **5**(1): 85-91.
3. Grabowski MK, Lessler J, Redd AD, et al. The role of viral introductions in sustaining community-based HIV epidemics in rural Uganda: evidence from spatial clustering, phylogenetics, and egocentric transmission models. *PLoS medicine* 2014; **11**(3): e1001610.
4. Kagaayi J, Gray RH, Whalen C, et al. Indices to measure risk of HIV acquisition in Rakai, Uganda. *PloS one* 2014; **9**(4): e92015.
5. Kiwanuka N, Laeyendecker O, Quinn TC, et al. HIV-1 subtypes and differences in heterosexual HIV transmission among HIV-discordant couples in Rakai, Uganda. *Aids* 2009; **23**(18): 2479-84.
6. Kouyoumdjian FG, Calzavara LM, Bondy SJ, et al. Intimate partner violence is associated with incident HIV infection in women in Uganda. *Aids* 2013; **27**(8): 1331-8.
7. Mathur S, Wei Y, Zhong X, et al. Partner Characteristics Associated With HIV Acquisition Among Youth in Rakai, Uganda. *Journal of acquired immune deficiency syndromes (1999)* 2015; **69**(1): 75-84.
8. Matovu JK, Gray RH, Kiwanuka N, et al. Repeat voluntary HIV counseling and testing (VCT), sexual risk behavior and HIV incidence in Rakai, Uganda. *AIDS and behavior* 2007; **11**(1): 71-8.
9. Nalugoda F, Guwatudde D, Bwaninka JB, et al. Marriage and the risk of incident HIV infection in Rakai, Uganda. *Journal of acquired immune deficiency syndromes (1999)* 2014; **65**(1): 91-8.
10. Santelli JS, Edelstein ZR, Mathur S, et al. Behavioral, biological, and demographic risk and protective factors for new HIV infections among youth in Rakai, Uganda. *Journal of acquired immune deficiency syndromes (1999)* 2013; **63**(3): 393-400.
11. Santelli JS, Edelstein ZR, Wei Y, et al. Trends in HIV acquisition, risk factors and prevention policies among youth in Uganda, 1999-2011. *Aids* 2015; **29**(2): 211-9.
12. Tobian AA, Ssempijja V, Kigozi G, et al. Incident HIV and herpes simplex virus type 2 infection among men in Rakai, Uganda. *Aids* 2009; **23**(12): 1589-94.
13. Wagman JA, Gray RH, Campbell JC, et al. Effectiveness of an integrated intimate partner violence and HIV prevention intervention in Rakai, Uganda: analysis of an intervention in an existing cluster randomised cohort. *The Lancet Global health* 2015; **3**(1): e23-33.
14. Wawer MJ, Makumbi F, Kigozi G, et al. Circumcision in HIV-infected men and its effect on HIV transmission to female partners in Rakai, Uganda: a randomised controlled trial. *Lancet (London, England)* 2009; **374**(9685): 229-37.
15. Zablotska IB, Gray RH, Serwadda D, et al. Alcohol use before sex and HIV acquisition: a longitudinal study in Rakai, Uganda. *Aids* 2006; **20**(8): 1191-6.
16. Gregson S, Mushati P, Grusin H, et al. Social capital and women's reduced vulnerability to HIV infection in rural Zimbabwe. *Population and development review* 2011; **37**(2): 333-59.

17. Gregson S, Nyamukapa C, Schumacher C, et al. Evidence for a contribution of the community response to HIV decline in eastern Zimbabwe? *AIDS care* 2013; **25 Suppl 1**: S88-96.
18. Gregson S, Nyamukapa C, Schumacher C, et al. Did national HIV prevention programs contribute to HIV decline in Eastern Zimbabwe? Evidence from a prospective community survey. *Sexually transmitted diseases* 2011; **38**(6): 475-82.
19. Mundandi C, Vissers D, Voeten H, Habbema D, Gregson S. No difference in HIV incidence and sexual behaviour between out-migrants and residents in rural Manicaland, Zimbabwe. *Tropical medicine & international health : TM & IH* 2006; **11**(5): 705-11.
20. Barnighausen T, Hosegood V, Timaeus IM, Newell ML. The socioeconomic determinants of HIV incidence: evidence from a longitudinal, population-based study in rural South Africa. *Aids* 2007; **21 Suppl 7**: S29-38.
21. Harling G, Newell ML, Tanser F, Barnighausen T. Partner Age-Disparity and HIV Incidence Risk for Older Women in Rural South Africa. *AIDS and behavior* 2015; **19**(7): 1317-26.
22. Tanser F, Barnighausen T, Hund L, Garnett GP, McGrath N, Newell ML. Effect of concurrent sexual partnerships on rate of new HIV infections in a high-prevalence, rural South African population: a cohort study. *Lancet (London, England)* 2011; **378**(9787): 247-55.
23. Biraro S, Ruzagira E, Kamali A, Whitworth J, Grosskurth H, Weiss HA. HIV-1 transmission within marriage in rural Uganda: a longitudinal study. *PloS one* 2013; **8**(2): e55060.
24. Ruzagira E, Wandiembe S, Abaasa A, et al. HIV incidence and risk factors for acquisition in HIV discordant couples in Masaka, Uganda: an HIV vaccine preparedness study. *PloS one* 2011; **6**(8): e24037.
25. Cawley C, Wringe A, Slaymaker E, et al. The impact of voluntary counselling and testing services on sexual behaviour change and HIV incidence: observations from a cohort study in rural Tanzania. *BMC infectious diseases* 2014; **14**: 159.
26. Hargreaves JR, Morison LA, Kim JC, et al. Characteristics of sexual partnerships, not just of individuals, are associated with condom use and recent HIV infection in rural South Africa. *AIDS care* 2009; **21**(8): 1058-70.
27. Wand H, Ramjee G. Combined impact of sexual risk behaviors for HIV seroconversion among women in Durban, South Africa: implications for prevention policy and planning. *AIDS and behavior* 2011; **15**(2): 479-86.
28. Kiwanuka N, Ssetaala A, Nalutaaya A, et al. High incidence of HIV-1 infection in a general population of fishing communities around Lake Victoria, Uganda. *PloS one* 2014; **9**(5): e94932.
29. Mermin J, Musinguzi J, Opio A, et al. Risk factors for recent HIV infection in Uganda. *Jama* 2008; **300**(5): 540-9.
30. Seeley J, Nakiyingi-Miiro J, Kamali A, et al. High HIV incidence and socio-behavioral risk patterns in fishing communities on the shores of Lake Victoria, Uganda. *Sexually transmitted diseases* 2012; **39**(6): 433-9.
31. Kumwenda N, Hoffman I, Chirenje M, et al. HIV incidence among women of reproductive age in Malawi and Zimbabwe. *Sexually transmitted diseases* 2006; **33**(11): 646-51.
