# Supplementary material for: Individual and population-level risk factors for new HIV infections among adults in Eastern and Southern Africa
Source: Nat Commun. 2026 Jan 6;17:1195. doi: 10.1038/s41467-025-67966-0 (PMC12858868; doi:10.1038/s41467-025-67966-0)
Supplement: Supplementary file 2 — Description of Additional Supplementary Files [file 41467_2025_67966_MOESM2_ESM.pdf]

Description of Additional Supplementary Files

Supplementary Data 1: Distribution of person years by socio-demographic characteristics and sexual behaviour among the men in each study for whom there is information, as a percentage of the total time spent in each age group.

Supplementary Data 2: Distribution of person years by socio-demographic characteristics and sexual behaviour among the women in each study for whom there is information, as a percentage of the total time spent in each age group

Supplementary Data 3: Crude HR for HIV incidence in each study among women aged 15-24

Supplementary Data 4: Crude HR for HIV incidence in each study among women aged 25-49

Supplementary Data 5: Crude HR for HIV incidence in each study among men aged 15-24

Supplementary Data 6: Crude HR for HIV incidence in each study among men aged 25-49

Supplementary Data 7: Adjusted HR for HIV incidence among women aged 15-24 from four multivariate models

Supplementary Data 8: Adjusted HR for HIV incidence among women aged 25-49 from four multivariate models

Supplementary Data 9: Adjusted HR for HIV incidence among men aged 15-25 from four multivariate models

Supplementary Data 10: Adjusted HR for HIV incidence among men aged 25-49 from four multivariate models

Supplementary Data 11: Adjusted HR for selected risk factors for women in Karonga, Kisesa and Rakai including measures of partnership dynamics, by age for 2005-16 inclusive

Supplementary Data 12: Adjusted HR for selected risk factors for men in Karonga, Kisesa and Rakai including measures of partnership dynamics, by age for 2005-16 inclusive

Supplementary Data 13: Adjusted HR for selected risk factors for 2013-16 only, based on data from Kisesa, Masaka, Rakai, uMkhanyakude, Kisumu and Ifakara
