# Supplementary material for: Individual and population-level risk factors for new HIV infections among adults in Eastern and Southern Africa
Source: Nat Commun. 2026 Jan 6;17:1195. doi: 10.1038/s41467-025-67966-0 (PMC12858868; doi:10.1038/s41467-025-67966-0)
Supplement: Supplementary file 4 — Reporting Summary [file 41467_2025_67966_MOESM4_ESM.pdf]

nature portfolio

Corresponding author(s): Emma Slaymaker

Last updated by author(s): Nov 27, 2025

Reporting Summary

Nature Portfolio wishes to improve the reproducibility of the work that we publish. This form provides structure for consistency and transparency in reporting. For further information on Nature Portfolio policies, see our Editorial Policies and the Editorial Policy Checklist.

Statistics

For all statistical analyses, confirm that the following items are present in the figure legend, table legend, main text, or Methods section.

n/a	Confirmed	
☐	☒	The exact sample size (*n*) for each experimental group/condition, given as a discrete number and unit of measurement
☒	☐	A statement on whether measurements were taken from distinct samples or whether the same sample was measured repeatedly
☐	☒	The statistical test(s) used AND whether they are one- or two-sided<br>*Only common tests should be described solely by name; describe more complex techniques in the Methods section.*
☐	☒	A description of all covariates tested
☒	☐	A description of any assumptions or corrections, such as tests of normality and adjustment for multiple comparisons
☐	☒	A full description of the statistical parameters including central tendency (e.g. means) or other basic estimates (e.g. regression coefficient) AND variation (e.g. standard deviation) or associated estimates of uncertainty (e.g. confidence intervals)
☐	☒	For null hypothesis testing, the test statistic (e.g. *F*, *t*, *r*) with confidence intervals, effect sizes, degrees of freedom and *P* value noted<br>*Give P values as exact values whenever suitable.*
☒	☐	For Bayesian analysis, information on the choice of priors and Markov chain Monte Carlo settings
☒	☐	For hierarchical and complex designs, identification of the appropriate level for tests and full reporting of outcomes
☒	☐	Estimates of effect sizes (e.g. Cohen's *d*, Pearson's *r*), indicating how they were calculated

Our web collection on statistics for biologists contains articles on many of the points above.

Software and code

Policy information about availability of computer code

Data collection	This is secondary analysis of data collected in 8 autonomous studies over a long period of time. Some data collection was paper-based and some was electronic; the software used by each data collection round in each study is not available.
Data analysis	No custom algorithms or software were used for this analysis. Stata version 18.0 was used for the analysis.

For manuscripts utilizing custom algorithms or software that are central to the research but not yet described in published literature, software must be made available to editors and reviewers. We strongly encourage code deposition in a community repository (e.g. GitHub). See the Nature Portfolio guidelines for submitting code & software for further information.

Data

Policy information about availability of data

All manuscripts must include a data availability statement. This statement should provide the following information, where applicable:

- Accession codes, unique identifiers, or web links for publicly available datasets
- A description of any restrictions on data availability
- For clinical datasets or third party data, please ensure that the statement adheres to our policy

A subset of the ALPHA harmonised incidence data used in this study for Karonga, Kisesa, Manicaland and uMkhanyakude have been deposited in the DataFirst database under accession codes mwi-alpha-him-karonga-2002-2017-v1 (Karonga) , tza-alpha-himk-1996-2016-v1 (Kisesa), zwe-alpha-himm-1995-2016-v1 (Manicaland), zaf-alpha-himu-2000-2016-v1 (uMkhanyakude) (https://www.datafirst.uct.ac.za/dataportal/index.php/collections/ALPHA). The data are available to

bona fide researchers under licensed access via DataFirst. The full set of data collected for this paper are individual participant data that cannot be anonymised whilst maintaining the information needed for this analysis. The study datasets used for this analysis (participant data with identifiers and accompanying data dictionaries) can be requested directly from the individual studies (see https://alpha.lshtm.ac.uk/people/). Requests will be reviewed by each study's data access committee according to their respective criteria for access.

Research involving human participants, their data, or biological material

Policy information about studies with human participants or human data. See also policy information about sex, gender (identity/presentation), and sexual orientation and race, ethnicity and racism.

Reporting on sex and gender	Referred to sex throughout.<br>Sex was a mix of self-reported, reported by a proxy respondent or assigned by an interview. None of the primary studies has systematically recorded how this was done over the lifetime of these studies, the earliest of which started in 1989.<br>We have disaggregated all results by sex and given the numbers of participants.
Reporting on race, ethnicity, or other socially relevant groupings	none
Population characteristics	Members of the general population who live in one of the study areas. We used data on time under observation when the participants were aged between 15 and 49. The incidence cohort used in this analysis required people to have had a negative HIV test and at least one subsequent HIV test. There were no other restrictions.
Recruitment	All residents in the study areas were eligible (with slight modifications in Manicaland and Kisumu) and invited to participate. Participation is high, though it varies between the studies, around 60% of people have contributed data.
Ethics oversight	LSHTM ethics committee approved this study. Each study also had its own approvals.

Note that full information on the approval of the study protocol must also be provided in the manuscript.

Field-specific reporting

Please select the one below that is the best fit for your research. If you are not sure, read the appropriate sections before making your selection.

☐ Life sciences ☒ Behavioural & social sciences ☐ Ecological, evolutionary & environmental sciences

For a reference copy of the document with all sections, see nature.com/documents/nr-reporting-summary-flat.pdf

Behavioural & social sciences study design

All studies must disclose on these points even when the disclosure is negative.

Study description	Secondary analysis of harmonised quantitative data on HIV incidence from 8 observational longitudinal studies
Research sample	Study sample consists of men and women aged 15-49 who were resident in the study area of 8 independently run longitudinal, population-based studies which collected HIV data from people living the study areas in 6 sub-Saharan African countries (Kenya, Malawi, South Africa, Tanzania, Uganda and Zimbabwe). People were eligible if they first tested negative for HIV and had a least one subsequent HIV test.<br>Data were harmonised by the ALPHA Network (https://alpha.lshtm.ac.uk/) and represent all the longitudinal population-based data on HIV for sub-Saharan Africa outside of trial settings. They are not nationally representative but are representative of the local area.<br>The rationale for this sample was twofold: firstly these are all the population-based studies in the region with directly observed data on incident HIV and the data cover the maximum period for which these data were available. Secondly the age range was chosen to maximise comparability across time and between studies as most had not conducted HIV testing for participants older than 49 in at least some rounds.
Sampling strategy	This is secondary analysis of studies that were originally established to estimate HIV incidence in each population. Each study is based on regularly updated census rounds and participants are not sampled- all residents are invited to participate.
Data collection	Data collection methods for each study are detailed in the cohort profiles referenced in the paper. It would be impossible to describe them all here because they change between rounds and are different for each study.
Timing	The data in this paper cover 2005 to 2016. The data collection that underlies these data spans a much longer period. HIV data collection for each study for the data used here: Ifakara 2012-2015; Karonga 2006-2012; Kisesa 1994-2016; Kisumu 2012-2016; Manicaland 1998-2012; Masaka 1989-2016; Rakai 2000-2016; uMkhanyakude 2003-2016.
Data exclusions	Across all the studies 159,296 people were enumerated at least one. 41,402 were excluded from this analysis: 14,323 were living with HIV throughout the whole time under observation and 27,079 were not followed up for long enough to be offered two HIV tests. Of the 117,894 who were eligible, 18,541 did not take two HIV tests leaving 99,353 people in the analysis which was 84% of those eligible for this study.

Non-participation	There are two possible points of drop out- those living in the area for a short time and those who did not participate in HIV testing. The reasons for these are not available. Short residence is not likely to be connected to study participation. Not taking HIV tests might be but there is no data to know how it could be related and this would be different between studies.
Randomization	no allocation

Reporting for specific materials, systems and methods

We require information from authors about some types of materials, experimental systems and methods used in many studies. Here, indicate whether each material, system or method listed is relevant to your study. If you are not sure if a list item applies to your research, read the appropriate section before selecting a response.

Materials & experimental systems

n/a	Involved in the study
☒	☐ Antibodies
☒	☐ Eukaryotic cell lines
☒	☐ Palaeontology and archaeology
☒	☐ Animals and other organisms
☒	☐ Clinical data
☒	☐ Dual use research of concern
☒	☐ Plants

Methods

n/a	Involved in the study
☒	☐ ChIP-seq
☒	☐ Flow cytometry
☒	☐ MRI-based neuroimaging

Plants

Seed stocks	*Report on the source of all seed stocks or other plant material used. If applicable, state the seed stock centre and catalogue number. If plant specimens were collected from the field, describe the collection location, date and sampling procedures.*
Novel plant genotypes	*Describe the methods by which all novel plant genotypes were produced. This includes those generated by transgenic approaches, gene editing, chemical/radiation-based mutagenesis and hybridization. For transgenic lines, describe the transformation method, the number of independent lines analyzed and the generation upon which experiments were performed. For gene-edited lines, describe the editor used, the endogenous sequence targeted for editing, the targeting guide RNA sequence (if applicable) and how the editor was applied.*
Authentication	*Describe any authentication procedures for each seed stock used or novel genotype generated. Describe any experiments used to assess the effect of a mutation and, where applicable, how potential secondary effects (e.g. second site T-DNA insertions, mosiacism, off-target gene editing) were examined.*
